# 708 Serratia infections in burn care

**DOI:** 10.1093/jbcr/irac012.262

**Published:** 2022-03-23

**Authors:** Alan D Rogers, David L Wallace

**Affiliations:** Ross Tilley Burn Centre, Toronto, Ontario; University of California, Davis, Sacramento, California

## Abstract

**Introduction:**

Burn wound infections, ventilator associated pneumonia, line sepsis and urinary tract infections are common in patients with major burn injuries, and remain prominent causes of morbidity and mortality. Within the spectrum of organisms responsible for infections, some are more common, while others might be relatively more virulent. This study sought to determine the impact of Serratia infections within the context of a verified regional burn centre.

**Methods:**

All patients admitted with a diagnosis of burn injury who developed an infection with Serratia Marcescens in the six years between 1 January 2015 and 31 December 2020, from any site, were included in the study. Data collected included demographic details, mechanism and extent of the burn, as well as the clinical course and complications.

**Results:**

Twenty two patients were included in the study, with a mean age of 46.5 years (range 27-70). Most had at least one significant co-morbidity. The mean burn size was 28% TBSA (range 2% - 71%), nine sustained inhalation injury and 13 required mechanical ventilation. Most patients underwent several surgeries (mean 3.4, range 1-9). The mean duration of hospital stay was 32.2 days (range 8-65), or 1.8 days per percentage burn (range 0.73 -4). For those who died, the mean number of days from admission to diagnosis of Serratia infection was 3.8 days (range 2-7), as against 10.11 days (range 1-27) for survivors. Eight of the first cultures were from sputum, 11 wounds, 4 blood cultures, and 1 from the urine. No significant resistant strains were identified, and all patients received timely and appropriate antibiotic therapy. Five of the patients died.

**Conclusions:**

Patients with major burn injuries are especially vulnerable to morbidity and mortality should they develop a systemic Serratia infection early in their hospital stay. Awareness of the natural history of these infectious episodes may improve the directed therapy required to improve outcomes.

